# Pharmacokinetics and Pharmacodynamics of T-Cell Bispecifics in the Tumour Interstitial Fluid

**DOI:** 10.3390/pharmaceutics13122105

**Published:** 2021-12-07

**Authors:** Miro Julian Eigenmann, Tine Veronica Karlsen, Marek Wagner, Olav Tenstad, Tina Weinzierl, Tanja Fauti, Hans Peter Grimm, Trude Skogstrand, Christian Klein, Johannes Sam, Pablo Umana, Marina Bacac, Helge Wiig, Antje-Christine Walz

**Affiliations:** 1 Roche Pharma Research and Early Development, Roche Innovation Center Basel, F. Hoffmann-La Roche Ltd., 4070 Basel, Switzerland; hans_peter.grimm@roche.com (H.P.G.); antje-christine.walz@roche.com (A.-C.W.); 2 Department of Biomedicine, University of Bergen, N-5020 Bergen, Norway; tine.karlsen@uib.no (T.V.K.); marek.wagner@biomed.uib.no (M.W.); olav.tenstad@uib.no (O.T.); trude.skogstrand@uib.no (T.S.); helge.wiig@uib.no (H.W.); 3 Roche Pharma Research and Early Development, Roche Innovation Center Zurich, 8952 Schlieren, Switzerland; tina.weinzierl@roche.com (T.W.); tanja.fauti@roche.com (T.F.); christian.klein.ck1@roche.com (C.K.); johannes.sam@roche.com (J.S.); pablo.umana@roche.com (P.U.); marina.bacac@roche.com (M.B.)

**Keywords:** T-cell bispecifics, pharmacokinetics, tumour uptake, cytokines, PBPK modelling, interstitial space

## Abstract

The goal of this study is to investigate the pharmacokinetics in plasma and tumour interstitial fluid of two T-cell bispecifics (TCBs) with different binding affinities to the tumour target and to assess the subsequent cytokine release in a tumour-bearing humanised mouse model. Pharmacokinetics (PK) as well as cytokine data were collected in humanised mice after iv injection of cibisatamab and CEACAM5-TCB which are binding with different binding affinities to the tumour antigen carcinoembryonic antigen (CEA). The PK data were modelled and coupled to a previously published physiologically based PK model. Corresponding cytokine release profiles were compared to in vitro data. The PK model provided a good fit to the data and precise estimation of key PK parameters. High tumour interstitial concentrations were observed for both TCBs, influenced by their respective target binding affinities. In conclusion, we developed a tailored experimental method to measure PK and cytokine release in plasma and at the site of drug action, namely in the tumour. Integrating those data into a mathematical model enabled to investigate the impact of target affinity on tumour accumulation and can have implications for the PKPD assessment of the therapeutic antibodies.

## 1. Introduction

Immune modulatory drugs are an important pillar for anticancer treatment [1]. Therapeutic antibodies, which are often used for immunotherapy, have an uneven distribution in plasma and tissue sub compartments [2,3,4,5]. For the development and evaluation of therapeutic antibodies it is critical to understand how much of the administered antibody reaches and binds to its therapeutic target and subsequently elicits the therapeutic effect [6,7]. A combination of preclinical in vitro and in vivo data together with mathematical modelling is commonly used in order to define a safe and efficacious human dose and dosing schedule. However, translation based on preclinical in vivo data can be limited in cases lacking cross-reactivity of the antibody or relevant differences in target expression in preclinical animals [8,9]. In such cases, the ability of a mathematical model to predict the distribution of an antibody to the target site in vivo represents a crucial aspect for in vitro to in vivo translation. When the therapeutic target is expressed in a tissue or solid tumour, the interstitial space is the most relevant compartment where therapeutic antibodies encounter their target [10]. Direct and quantitative measurements of antibody drug levels in the tumour interstitial fluid, whilst accounting for free and bound drug, are experimentally challenging and various experimental methods were previously reviewed in literature [11,12]. The tissue centrifugation method is employed to isolate tissue interstitial fluid from tissue biopsies, and it has been successfully evaluated for several tissues (skin, muscle, tumour, tendon) [11,13,14,15]. As such, it is a valuable tool to investigate the pharmacokinetic (PK) of therapeutic antibodies in the interstitial fluid space [14] and to relate it to its pharmacodynamic (PD) effects.

Cibisatamab (CEA-TCB) and the analogous CEACAM5-TCB are two 2 + 1 CD3 engaging T-cell bispecific antibodies (TCBs) with a molecular weight of 194 kDa [16,17]. Both molecules are directed against the carcinoembryonic antigen (CEA) [18],however, exhibit different monovalent binding affinities (16 nM and 0.09 nM for cibisatamab and CEACAM5-TCB respectively), different avidity effect and bind to distinct epitopes of CEA.

**Figure 1:**
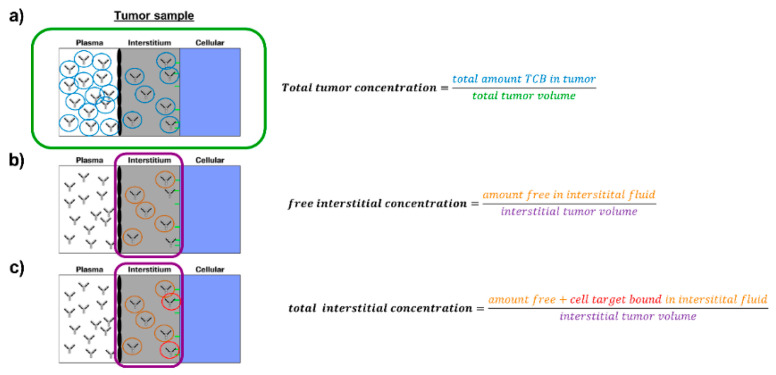
Schematic definitions of tumour-focused PK readouts: (**a**) Total tumour concentration (TTC), which relates the total amount of antibodies to the total tumour tissue sample volume, with no distinction of plasma, interstitial or cellular compartment, (**b**) free interstitial concentration (FIC), which represents what is directly measured by the centrifugation method, i.e., free antibodies specifically in the interstitial fluid space, (**c**) total interstitial concentration (TIC), which accounts for free and cell surface target-bound antibodies specifically in the interstitial fluid space.

Our goal in this work was to measure and compare the PK of those two TCBs in plasma, total tumour and tumour interstitial fluid in humanised NOD scid gamma (NSG) mice [19,20,21]. Tumour growth inhibition and cytokine release were assessed as subsequent PD readouts under TCB treatment. Cytokine release was then compared in tumour interstitial fluid and systemic plasma samples and additionally put in relation to corresponding in vitro data. We further provide granularity around measured and derived drug concentrations target site (total tumour concentration (TTC), free interstitial concentration (FIC), bound interstitial concentration (BIC) and total interstitial concentration (TIC)). The definition of those readouts, which are further illustrated in Figure 1, is important to interpret the results within this work and put them into broader a context. Finally, based on the generated pharmacokinetic data of the TCBs, we established a mathematical tumour uptake model and coupled it to a previously published physiologically based pharmacokinetic (PBPK) model [14]. Using this model, predictions can be performed for tumour and healthy tissues in order to evaluate the potential implication of tissue distribution on the efficacy and safety profile of those molecules, taking into consideration tissue dependent differences in target expression. With this, we believe to improve significantly the understanding and prediction of tumour interstitial PK for immunoglobulin G (IgG)-based antibodies and the impact of binding properties of targeted antibody therapeutics on tumour distribution and accumulation.

## 2. Materials and Methods

### 2.1. Ethical Approval

All animal studies were approved by the AAALAC International Accredited Animal Care and Use Program at the University of Bergen. The studies were conducted according to, and with approval of, the Norwegian State Commission for Laboratory Animals (approval # 18850, 1 November 2019), which are aligned with the European Convention for the Protection of Vertebrate Animals used for Experimental and Other Scientific Purposes and Council of Europe (ETS 123).

### 2.2. Study Overview

In this work, we performed several experimental studies and a mathematical modelling analysis. Furthermore, some studies were performed as preparation and prerequisite of a main biodistribution study. Therefore, a comprehensive overview across all studies, which are part of this work including their main features and purpose, are summarised in Supplementary Table S1. Details for each study are provided in the respective Methods section.

### 2.3. Animal Studies and Tumour Engrafting

All mice were housed under a 12:12 h light/dark cycle and received standard chow and water ad libitum, both before and during experiments. During invasive procedures, the animals were anesthetised with IsoFlo^®^Vet, 1.5% isoflurane in 100% O_2_ (Abbott Laboratories Ltd., Maidenhead, UK). The anaesthesia was monitored, and the body temperature was kept stable using a heating pad. At the end of the study, mice were euthanised under anaesthesia by excision of the heart.

Radiotracer studies were performed in 13 male NSG mice (12 weeks old, in house breeding) and the biodistribution study was conducted in 45 female humanised NSGTM mice engrafted with CD34+ hematopoietic stem cells (provided by The Jackson Laboratory, Bar Harbor, ME, USA). In those humanised NSG mice, human CD4+ and CD8+ T cells are present in circulation and tissues, offering a model in which effects of T-cell dependent immunotherapy can be studied [19,20]. When 34 weeks old (body weight ranging from 17–24 g), the mice were engrafted by subcutaneous injection of 1 million MKN45 tumour cells (DSMZ, Braunschweig, Germany) on the back of the mice. Before engraftment, the cells were cultured at 37 °C in 5% CO_2_ in RPMI-1640 culturing medium with 10% FBS (Sigma, St. Louis, MO, USA) and 1% GlutaMax. 100U/mL penicillin as well as 100mg/mL streptomycin (ThermoFisher scientific, Waltham, MA, USA) and plasmocin (InvivGEN, Toulouse, France) were added.

The investigators were blinded throughout the in vivo experiments and data acquisition and did not know the identity/characteristics of the compounds. In case of early termination of the mice, terminal measurements were taken and reported at time of termination.

### 2.4. Radiolabelled Probes and Quality Control

Human serum albumin (HSA) was used as plasma volume tracer and was labelled with ^125^Iodine using the iodogen method, which was previously described in detail [22,23]. Furthermore, 51Cr-EDTA (PerkinElmer Health Sciences B.V, Groningen, The Netherlands) served as an extracellular fluid tracer. For the biodistribution study, cibisatamab (20 mg/mL in 20 mM histidine/His-HCl, 240 mM sucrose, 10 mM methionine, 0.05% polysorbate 20 at pH 5.5) and CEACAM5-TCB (1.93 mg/mL in 20 mM Histidine, 140 mM NaCl at pH 6.0) (both provided by Roche) were also labelled with ^125^Iodine. In order to avoid impact of the labelling procedure on the pharmacological activity, the two T-cell bispecific antibodies were labelled by an indirect labelling approach. After labelling, the retention of the biological activity was confirmed and potentially unbound ^125^Iodine was removed prior to each injection. The labelling procedure and quality control is described in more detail in the supplementary material S1 and Figure S1 respectively.

### 2.5. Interstitial Fluid Isolation and Radiotracer Studies

The feasibility to isolate a representative interstitial fluid sample from tumour by centrifugation was assessed. About 13 mice were terminated to harvest tumour and a terminal plasma sample at 14 (*n* = 4), 17 (*n* = 4) or 20 days (*n* = 5) after tumour cell engraftment. Skin was harvested as reference sample. The tumour size and corresponding volume of isolated interstitial fluid was measured. The isolation of interstitial fluid from harvested tissue samples was based on a tissue centrifugation methodology, which has been previously described [13]. The tissue samples were transferred onto a mesh with a pore size of 15–20 μm in an Eppendorf tube and immediately centrifuged for 10 min at 424 g. The fluid sample at the bottom of the tube was collected and further analysed. In order to ensure that this is a good surrogate for native TIF and to assess the composition of the tumour sample, radiotracer studies were performed evaluating the plasma and extracellular fraction in those samples.

For the use as plasma volume tracer, ^125^Iodine-HSA was injected intravenously in a group of mice 17 days post-engraftment. After 5 min distribution time, the mice were euthanised allowing the large molecule tracer to homogeneously equilibrate in plasma while limiting the extravasation into the tissue space [24,25]. ^51^Cr-EDTA (1 million cpm/mouse) was used as an extracellular tracer in two separate groups of mice, either at day 14 or 20 post-engraftment. Before injection of the tracer, the kidneys of the mice were surgically tied off to limit renal excretion. An equilibration time of 60 min, before terminating the mice, allowed the tracer to equilibrate in all extracellular spaces (i.e., plasma and interstitial fluid). Normalising the counts in a harvested tissue sample with the counts in plasma allows deriving plasma and extracellular volume fraction in the respective sample, using the following equations:

(1) $$plasma\;fraction\;\left(fVpla\right)=\frac{125I\;\mathrm{count}}{g\;tumor}/\frac{125I\;\mathrm{count}}{\mathrm{mL}\;plasma}$$

(2) $$extracellular\;fluid\;fraction\;\left(fVec\right)=\frac{51\mathrm{Cr}\;\mathrm{counts}}{g\;sample}/\frac{151\mathrm{Cr}\;\mathrm{counts}}{\mathrm{mL}\;plasma}$$

(3) interstitial fluid fraction (fVint)=fVec−fVpla

The same approach was followed to evaluate the isolated fluid sample by comparing the counts in the isolated fluid to plasma. An undiluted interstitial fluid sample should have an extracellular fluid fraction close to 1.0 and a low plasma fraction, as previously reported in literature [13].

### 2.6. Biodistribution Study and Pharmacodynamic Assessment

Tumour size was monitored every other day using calliper measurements (π/6 × a^2^ × b). Upon reaching 200 mm^3^, three mice per time point were randomly assigned to each treatment group, untreated control (*n* = 9), 2.5 mg/kg cibisatamab (*n* = 18) or 2.5 mg/kg CEACAM5-TCB (*n* = 18). Unlabelled and labelled antibody was mixed at a given ratio and injected intravenously in the tail vein at a dose of 2.5 mg/kg with 4–6 million cpm per mouse. The mice were terminated at predefined time points, 1, 8, 24, 48, 96 or 240 h or 1, 48 and 240 h after dosing in case of the control group. Due to mortality of some mice, the earliest measured time point for CEACAM5-TCB was 8 h. In case of early termination (e.g., due to necrosis of the tumour), all terminal sampling was performed as described below and the time point of termination was reported and used for analysis. A terminal blood sample was collected from each mouse and tumours were excised and centrifuged as described above. The blood sample was centrifuged at 10,621× *g* in order to prepare a plasma sample. Total tumour samples, plasma and tumour interstitial fluid (TIF) were transferred to separate vials for γ-counting in order to assess the amount of compound in the respective sample. As the isolation of interstitial fluid by centrifugation often yields small sample volumes with a relatively high surface to volume ratio, all sample handling was done in a humidity chamber (100% relative humidity) in order to avoid evaporation from the sample [13]. The specific activity was used to derive the compound concentration in the samples. Half of the plasma and TIF fluid sample were frozen at −80 °C for cytokine analysis. The residual plasma fraction in harvested total tumour samples, which was measured during the radiotracer studies, was used in order to account for the amount of antibody in residual plasma of the tumour sample [26,27]. Furthermore, the isolated interstitial fluid sample from the tumour by centrifugation is not completely pure and therefore, the residual plasma- and extracellular fluid fraction in the isolated fluid sample was used to correct for the plasma- and intracellular fluid contamination in the sample and to derive the corrected free interstitial concentration.

### 2.7. Cytokine Measurement

Cytokine profiles were measured in plasma and TIF from the mice by Magnetic Luminex Performance Assay (R&D Systems, Minneapolis, MN, USA) for IL2, IL6, IL10, TNF-α, Granzyme B and IFN-γ according to the manufacturer’s description. In short, samples were prepared by adding calibrator diluent to 60 µL of sample fluid, achieving a two-fold dilution. Total of 50 µL standard, control and samples were each added in duplicates to the designated wells before microparticle cocktail was added to the plate and incubated for 2 h at room temperature on a plate shaker. After three washing steps, biotin-antibody cocktail was added and incubated for one hour before the addition of Streptavidin-PE and additional 30 min of incubation. Finally, Wash Buffer was added to each well and the plate was measured on a MagPix Luminex reader (Luminex, Austin, TX, USA). Duplicate readings were averaged, and the background mean fluorescence intensity (MFI) subtracted. Each sample was then compared to the standard curve of the respective analyte.

The in vivo cytokine data were then compared to respective in vitro data, which were collected in a corresponding in vitro system. The measurement of the in vitro cytokine release has been reported in more detail [28]. In short, MKN45 tumour cells were co-cultured with human peripheral blood mononuclear cells (PBMCs) and treated by adding a titration of concentrations of either cibisatamab (6–100,000 pM) or CEACAM5-TCB (1–20,000 pM) to the assay medium. Cytokine analysis was then performed using CBA kits (Becton Dickinson, Franklin Lakes, NJ, USA) according to manufacturer’s instructions at 24, 48, 72, 96 and 168 h. The measured IL6, IL2, IL10, TNFα, IFNγ concentrations were then compared with the in vivo cytokine data in tumour interstitial fluid.

### 2.8. Tumour Uptake Modelling

The experimental tumour uptake data were used in order to estimate underlying PK and tumour uptake parameters. The tumour uptake model was inspired by previously published work around tumour uptake modelling and performed in Matlab 2018a including the Simbiology toolbox (The MathWorks Inc., Natick, MA, USA). The plasma PK was described by a previously published PBPK model [14], which was then linked to the tumour uptake model. The PBPK model was adapted in two smaller aspects: (1) for simplification of the model, the plasma spaces (systemic and all tissues) were lumped together into one plasma compartment and consequently the blood flows were removed from the model [14,26]. We ensured that this change did not affect simulated PK outputs of the model (Supplementary Figure S2). As all parameters for the tissues in the model remained fixed to the originally published values, we added a “Rest of body” compartment to account for any additional peripheral distribution. The resulting model framework enables simulation of antibody PK in sub-compartments of different tissues.

**Figure 2:**
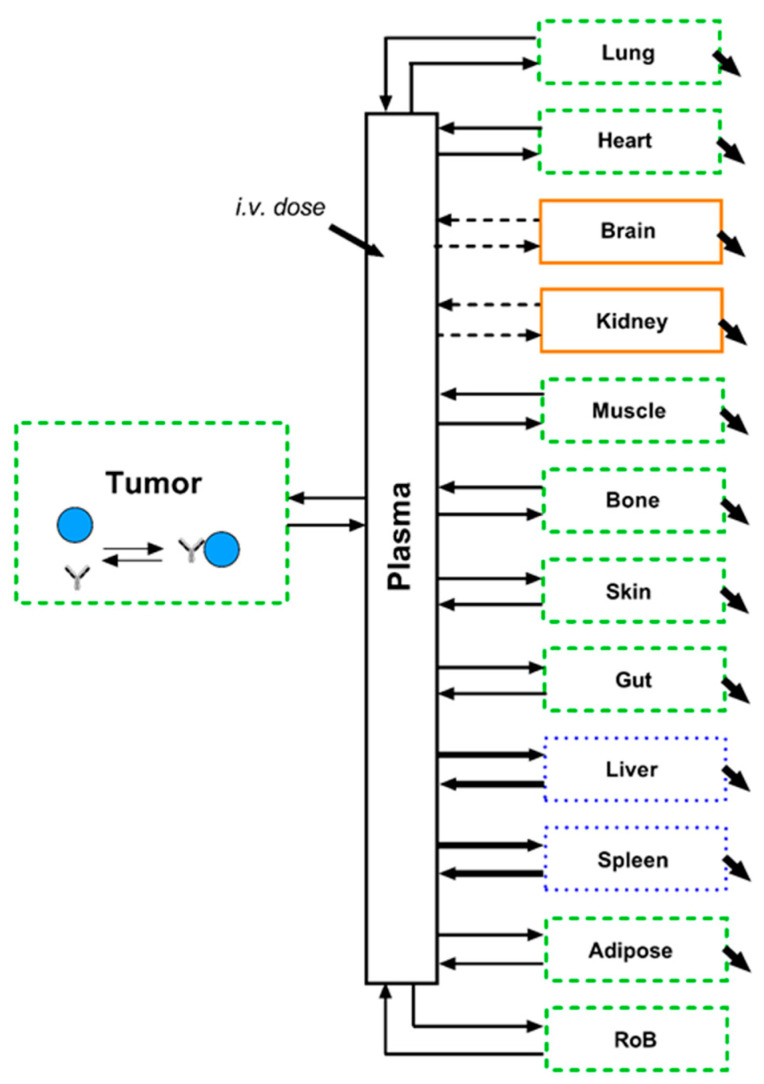
Schematic illustration of the final tumour uptake model linked to the PBPK model: The PBPK model contains three categories of organs, depending on the underlying vascular structure. Black arrows indicate rate constants for antibodies (i.e., distribution, binding, elimination). Blue dotted: organs with discontinuous capillaries and interstitial space assumed highly accessible for antibodies and in quick equilibrium with plasma space (bold arrows). Green dashed: organs with continuous capillaries and interstitial space accessible for antibodies with a distinct uptake and outflow for antibodies. Orange solid: organs with specialised capillaries (e.g., glomerular filter & blood brain barrier) and interstitial space largely non-accessible for antibodies (dashed arrows). Binding of antibodies to cell surface target on tumour cells included in the tumour interstitial compartment.

A graphical illustration of the final model structure is depicted in Figure 2. All parameters and equations related to other tissues (except tumour and rest of body) were kept according to the original publication of the underlying PBPK model [14]. The model equations are reported in the Supplementary Material (S2). Definitions of the model outputs can be found below:

The total tumour concentration of the antibody changing over time is defined as follows:

(4) $$TTC=fVpla_{tum}*C_{pla}+fVint_{tum}*Cint_{tot},$$

where *fVpla_tum_* is the residual plasma fraction in the tumour sample and *fVint_tum_* is the interstitial volume fraction in the tumour sample as measured in this work.

The free interstitial concentration of the antibody changing over time is defined as follows:

(5) $$FIC=\frac{Aint_{free}}{Vint_{tum}},$$

where *Aint_free_* is the unbound interstitial amount of antibody and *Vint_tum_* is the interstitial fluid volume in the tumour sample.

The bound interstitial concentration of the antibody changing over time is defined as follows:

(6) $$BIC=\frac{Aint_{bnd}}{Vint_{tum}},$$

where *Aint_bnd_* is the antibody concentration bound to the target in the interstitial space.

The total interstitial concentration of the antibody changing over time is defined as follows:

(7) $$TIC=\frac{Aint_{free}+Aint_{bnd}}{Vint_{tum}}$$

The biodistribution data of the two T-cell bispecific antibodies were fitted simultaneously with the same system parameters, while for each of the two compounds different drug specific parameter values were estimated. Estimated system parameters were: tumour uptake rate (Up_tum_), backflow rate from tumour (Out_tum_), uptake rate to rest of body (Up_RoB_), backflow rate from rest of body (Out_RoB_), plasma volume (Vpla) and tumour target expression (targ). The total tumour volume (TV) was fixed to 250 µL based on the measured tumour volume data under TCB treatment (Section 3.3). Fixing this allowed to simplify the model equation as the change in tumour volume would add an additional layer of complexity in the model. All other system parameters were fixed according to the previously published PBPK model [14]. Plasma clearance and binding affinity were the only drug-specific parameter, which were estimated from the data. As measure of accuracy, the estimation confidence intervals were derived for the respective parameters using the inversed Jacobian matrix from lsqnonlin and the nlparci function in Matlab. Furthermore, a local sensitivity analysis was performed in the Simbiology toolbox, evaluating the sensitivity of each fitted model readout (plasma PK, total tumour PK and free interstitial PK) to each of the estimated parameter. Additionally, the model performance was evaluated based on the accuracy of the parameter estimates (% CI), visual inspection of the model fit to the data and Goodness-of-Fit plots (observed vs. predicted).

### 2.9. Statistical Analysis

Statistical data analysis and plotting was performed in GraphPad Prism 8 (GraphPad Software, San Diego, CA, USA) and data were plotted as mean ± SD unless stated otherwise. When comparing the different treatment groups or readouts, a one way ANOVA was performed followed by a post hoc Bonferroni test. PD effects were compared based on AUCE differences. Differences with *p* < 0.05 were considered significant.

For the in vivo studies, the group sizes within one experiment were designed to be equal based on the number of available mice and the mice were randomly assigned to the various groups. The group sizes for the radiotracer studies in the NSG mice were defined based on prior experience with the employed methods and expected variability. The mice were randomly assigned to the assessed time points (14 (*n* = 4), 17 (*n* = 4) or 20 days (*n* = 5) after tumour engraftment). Due to prior experience, the terminal nature of the measurements and the fact that the radiotracer study was mainly a feasibility study to inform and optimise the following biodistribution study, the number of animals per group was kept to a minimum. No formal sample size analysis or statistical analysis between the individual groups was performed for this study. For the biodistribution study with subsequent PD evaluation, mice were each randomly assigned to the two treatment groups (*n* = 18) and to the vehicle group (*n* = 9). The vehicle group was smaller in number as less terminal time points were sampled for the PD readouts in the control group to define the baseline. Due to mortality of the mice, the group sizes had to be adjusted to *n* = 18 for the cibisatamab and *n* = 17 for the CEACAM5-TCB treatment group. As the measurements were terminal, three mice per treatment group were terminated per time point. In case of early termination of the mice, terminal measurements were taken and reported at time of termination.

## 3. Results

### 3.1. Isolated Fluid by Centrifugation Is a Surrogate for Tumour Interstitial Fluid

**Figure 3:**
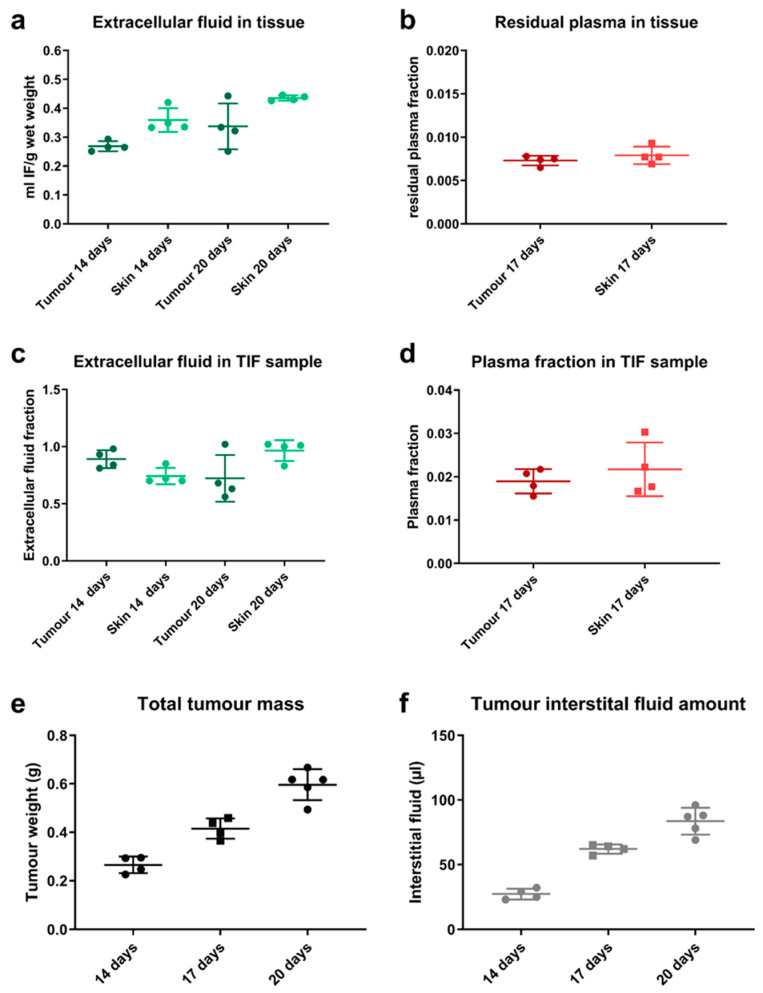
Composition of tissue sample and isolated fluid sample: (**a**) Extracellular volume fraction in tumour and skin sample as measured by ^51^Cr-EDTA distribution (1 h after injection) in mice; (**b**) residual plasma volume fraction in harvested tumour and skin tissue as measured by ^125^Iodine-HSA distribution (5 min after injection); (**c**) extracellular fluid fraction in the isolated fluid sample from centrifugation of skin and tumour samples and (**d**) plasma contamination, in terms of residual plasma volume fraction, in the isolated fluid sample after centrifugation; (**e**) weight of tumour samples harvested from mice at 14, 17 and 20 days post-engraftment and, (**f**) corresponding tissue fluid sample volumes, which were isolated by centrifugation. Individual data are presented, incl. mean ± SD (*n* = 4, *n* = 5 on day 20).

In the first step, we assessed if the tissue centrifugation method yields fluid samples of sufficient volume, which could be used as surrogate for native tumour interstitial fluid. In order to demonstrate this, the composition of harvested tumour (Figure 3a,b) and of the respective isolated tissue fluid samples after centrifugation (Figure 3c,d) was evaluated. As the centrifugation method was previously evaluated for skin, measurements in skin samples were used as quality control to evaluate the performance of the experimental method. The data from skin tissue were not used for any statistical comparison to values measured in tumour.

The extracellular tracer indicates an extracellular volume of 29% in tumour and 40% in skin (Figure 3a). Low levels of residual plasma were measured in tumour and skin tissue samples, 0.73 and 0.79% respectively (Figure 3b). The extracellular volume fraction in isolated fluid from tumours was 89% on day 14 after tumour engraftment and 66% with bigger tumours, 20 days after engraftment (Figure 3c). In isolated tumour tissue fluid, the plasma contamination was low at 1.9% (Figure 3d). This indicates a higher dilution of the isolated sample with intracellular fluid at higher tumour volumes. The tumour growth was relatively uniform in the mice, reaching a mean tumour weight of 265, 415 and 596 mg after 14, 17 and 20 days group respectively (Figure 3e). The isolated tissue fluid volume correlated with the respective tumour size, yielding 27, 62 and 84 µL respectively and corresponded to ~10–15% of the harvested tumour sample (Figure 3f). The values measured for skin were all in agreement with literature [14,25,29]. In summary, the data indicate that the isolated tissue fluid from MKN45 tumours can be used as a surrogate for interstitial fluid with minimal corrections for intracellular fluid dilution and plasma contamination in the isolated fluid sample.

### 3.2. High TCB Concentrations Observed in the Tumour Interstitial Fluid

**Figure 4:**
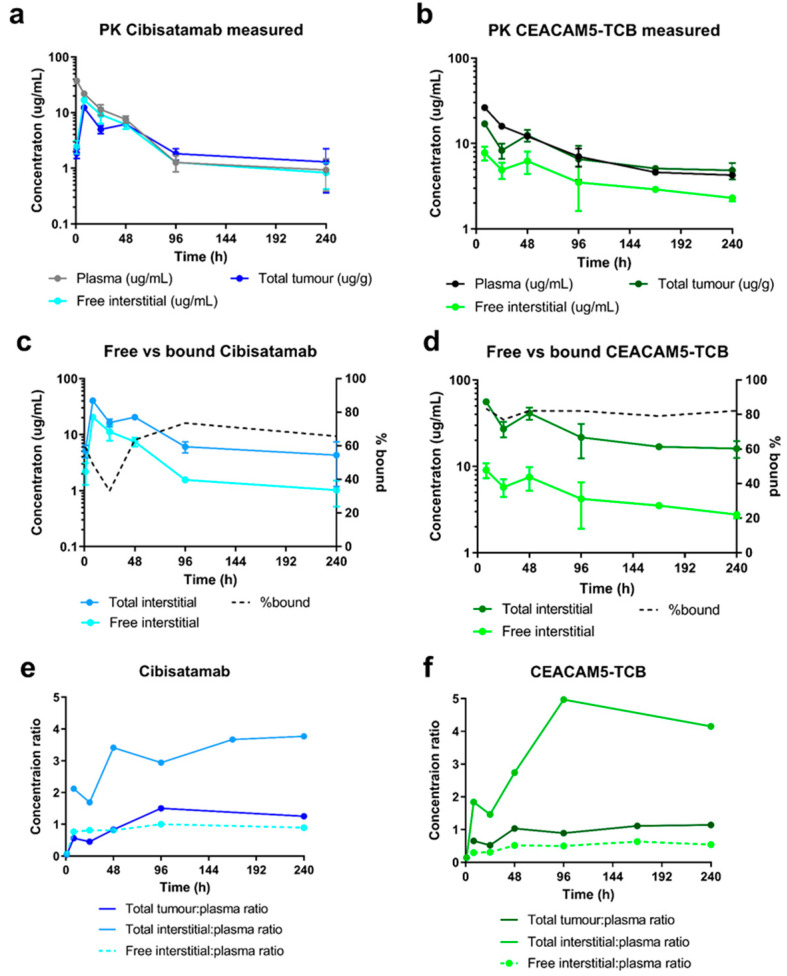
Measured or derived PK readouts based on the biodistribution study: plasma-, total tumour- and free interstitial PK profiles measured over 10 days after injection of a TCB dose of 2.5 mg/kg in the humanised NSG mice for (**a**) cibisatamab (*n* = 18) and (**b**) CEACAM5-TCB (*n* = 17), (**c**) measured free interstitial- compared to the derived total interstitial PK and the corresponding % of bound antibody for cibisatamab and (**d**) CEACAM5-TCB, (**e**) calculated total tumour: plasma- (dark), total interstitial: plasma (light) and free interstitial: plasma (dashed) concentration ratio for cibisatamab (blue) and (**f**) CEACAM5-TCB (green). At each time point three mice of each treatment group were terminated and measured and data are presented as mean ± SD.

A biodistribution study was performed in humanised NSG mice after intravenous (i.v.) injection of either cibisatamab or CEACAM5-TCB in order to investigate the distribution of the two TCBs into the tumour interstitial space and their accumulation therein due to target binding. The measured plasma PK showed a higher clearance for cibisatamab (0.043 mL/h) as compared with CEACAM5-TCB (0.015 mL/h). The free interstitial concentration in the tumour was slightly higher than the total tumour concentration for cibisatamab while the opposite was observed for CEACAM5-TCB, which might be due to the higher CEA binding affinity of CEACAM5-TCB (Figure 4a,b). Based on the total- and free interstitial tumour PK data, it was derived that most of the intratumoural antibodies were bound to their cell surface target, with ~65% for cibisatamab and 80% for CEACAM5-TCB respectively (Figure 4c,d). The comparison of total interstitial concentrations to total tumour concentrations illustrated that interpreting tissue PK only based on total tissue concentrations would lead to an underestimation of the actual interstitial concentrations for TCBs. This was also indicated by the calculated total tumour: plasma- and total interstitial: plasma concentration ratios, which were much higher when total interstitial concentrations are considered. The total interstitial: plasma ratio of CEACAM5-TCB was higher as compared to cibisatamab, 5- vs. 3-fold respectively (Figure 4e,f). Overall, all presented data indicated that high TCB concentrations were achieved in tumour interstitial fluid (TIF) after intravenous dosing and that tumour accumulation was further increased depending on the target binding affinity.

### 3.3. Strong Tumour Growth Inhibition Observed under TCB Treatment

**Figure 5:**
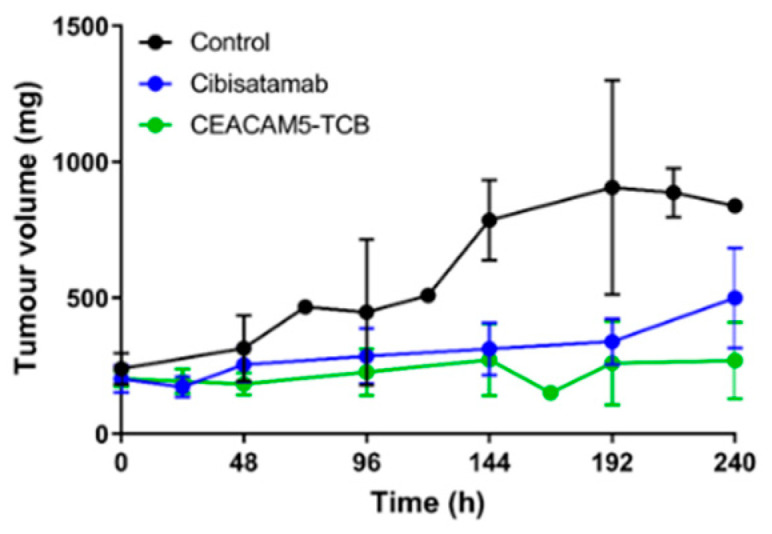
Tumour growth inhibition after single dose of TCB: Tumour volumes were reported by calliper measurements. Untreated tumour growth is shown for the vehicle group (black, *n* = 9) and tumour growth inhibition over 10 days after i.v. administration of 2.5 mg/kg of TCBs for cibisatamab (blue, *n* = 18) and CEACAM5-TCB (green, *n* = 17). Data are represented as mean ± SD. Cibisatamab and CEACAM5 treatment both showed significant treatment effects when compared to the control group (*p* = 0.004 and 0.001 respectively but no significant difference in between the two treatments at a dose of 2.5 mg/kg (*p* = 0.8). At each predefined time point, 3 mice were terminated for PK and cytokine assessment. If mice were euthanised prematurely due to necrosis of the tumour, the times could deviate from the protocol and the values were reported at time of termination.

In order to assess the antitumor efficacy of the two TCBs, the tumour growth was monitored in the vehicle- and TCB-treated mice over 10 days after dosing. For both compounds, strong tumour growth inhibition was observed already 4 days after treatment start. This was further corroborated by a one-way ANOVA for repeated measures (*p* = 0.001) with the post hoc Bonferroni’s test (performed in GraphPad PRISM version 8.0) showing significant differences between the treated and control group, cibisatamab vs. control (*p* = 0.004, mean Area under the curve of effect (AUCE) difference = 75,038 mm^3^/h), CEACAM5-TCB vs. control (*p* = 0.001, mean AUCE difference = 91,080 mm^3^/h). The difference between CEACAM5-TCB vs. cibisatamab treatment at a dose of 2.5 mg/kg was not significant in that study (*p* = 0.8) and both showed very strong anti-tumour effect with a lower mean AUCE of 16,042 mm^3^/h in case of CEACAM5-TCB. Under treatment, the low tumour volume was maintained over the course of the 10 days, despite no additional TCB administration (Figure 5).

### 3.4. Higher Cytokine Levels Observed in Tumour Microenvironment as Compared to Plasma

As part of the investigation of subsequent local and systemic PD effects under cibisatamab and CEACAM5-TCB treatment, cytokine concentrations were measured in TIF and plasma. Those data were used to compare local and systemic effects on cytokine release and as comparison to measured cytokine release data in vitro.

**Figure 6:**
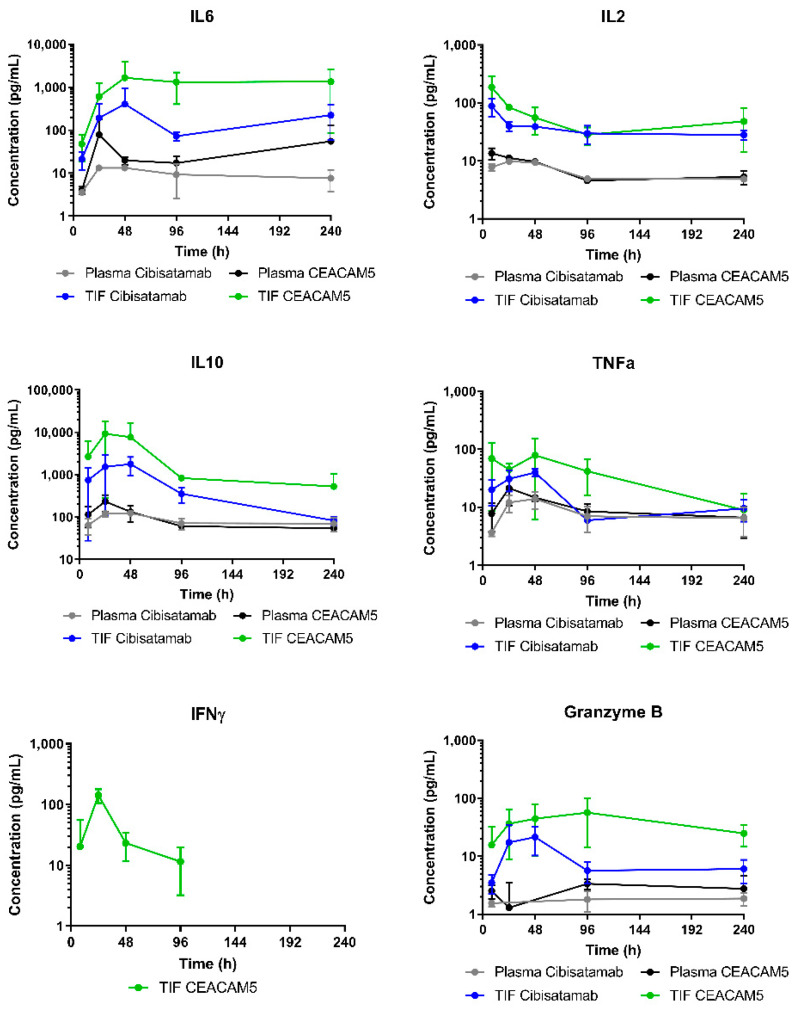
Cytokine profiles in plasma and TIF after single dose TCB i.v. injection: Cytokine (IL2, IL6, IL10, TNFα, IFNγ and Granzyme B) concentration profiles during the first 10 days after 2.5 mg/kg TCB injection, captured in plasma and in isolated TIF for cibisatamab (*n* = 18, grey and blue respectively) and CEACAM5-TCB (*n* = 17, black and green respectively). Data are represented as mean ± SD. IL2 and Granzyme B showed significantly higher levels in TIF vs. plasma (*p* < 0.009 and <0.03, respectively). Despite a consistent trend across all cytokines, the other cytokines showed no statistically significant differences, neither in TIF vs. plasma nor between the two TCB treatments.

**Figure 7:**
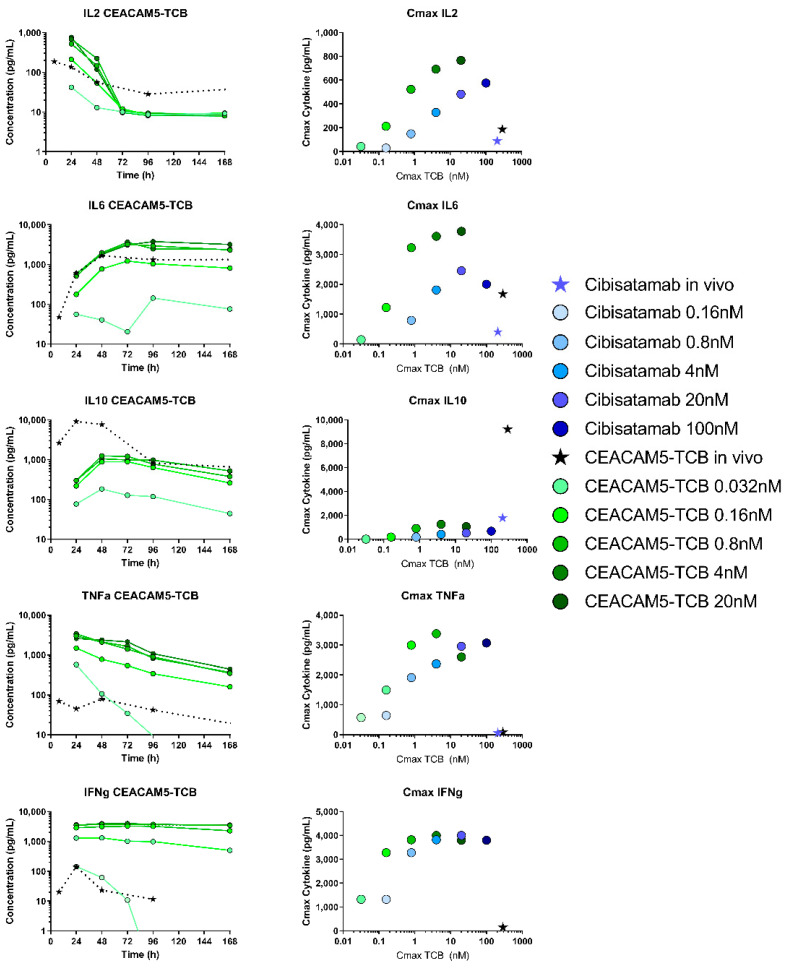
Comparison of cytokine release under TCB treatment in vitro and in vivo: Cytokine concentration profiles during the first 7 days after TCB injection, which corresponds to the maximal duration measured in vitro (data only shown representatively for CEACAM5-TCB) exhibit similar kinetics in vitro (green) as compared to in vivo (dose of 2.5 mg/kg) (black) (left panel). Further comparison of the correlation of Cmax of the drug and the observed Cmax of the cytokines in vitro and in vivo under cibisatamab (blue) and CEACAM5-TCB (green) treatment. For visual clarity, the data are shown as mean without SD and only the higher range of tested in vitro concentrations are depicted in comparison to the high in vivo Cmax.

As indicated in Figure 6, all measured cytokines levels were in general 1–2 order of magnitude higher in TIF than in plasma. CEACAM5-TCB treatment led to a higher mean of local cytokine release in TIF and in vitro as compared to cibisatamab, which was most apparent for IL6, Granzyme B and IL10. For TNFα and IL2, the higher effect of CEACAM5-TCB was mainly observed at early time points and differences decreased over the course of the 10 days. All cytokines were detected up to 10 days in TIF and plasma, with the exception of IFNγ, which could only be detected under CEACAM5-TCB treatment in TIF and up to 96 h and was below the limit of quantification otherwise. IL2 and Granzyme B showed significant differences of levels in TIF versus plasma (*p* < 0.009 and *p* < 0.03, respectively). Despite a pronounced difference in mean AUCE and a consistent trend across all cytokines, the statistical analysis on the other cytokines individually showed no significance in AUCE difference between TIF and plasma (*p*-values between 0.057–0.11), which is probably due to the relatively high inter-individual variability in the measurements. Interestingly, the cytokine release kinetics observed in TIF was similar to the kinetics observed in an in vitro co-culture assay with the same MKN45 tumour target cells and PBMCs (Figure 7). However, at comparable TCB concentrations, the absolute cytokine levels observed in TIF were lower as compared to in vitro. Only for IL10, the observed initial peak was more pronounced and earlier in vivo as compared to in vitro (Figure 7).

### 3.5. Tumour Uptake Modelling Allowed a Good Description of the Biodistribution Data and a Precise Quantification of Underlying Tumour Uptake Parameters

In addition to the insights generated based on the observed biodistribution data, we aimed to quantify underlying PK parameters using a mathematical model. A tumour uptake model was built based on the previously published work [30,31] and adapted in order to describe and fit the biodistribution data. The antibody distribution into the “Rest of Body” compartment, CEA target expression level, monovalent target binding affinity and distribution rates into and out of the tumour were estimated as part of the refinement process. Furthermore, the tumour uptake model was linked to an existing PBPK model [14]. Once set up, such a mathematical model framework could then be used to simulate and predict the PK for therapeutic antibodies in various tissues of interest, also considering the respective target expression levels and target binding properties.

**Figure 8:**
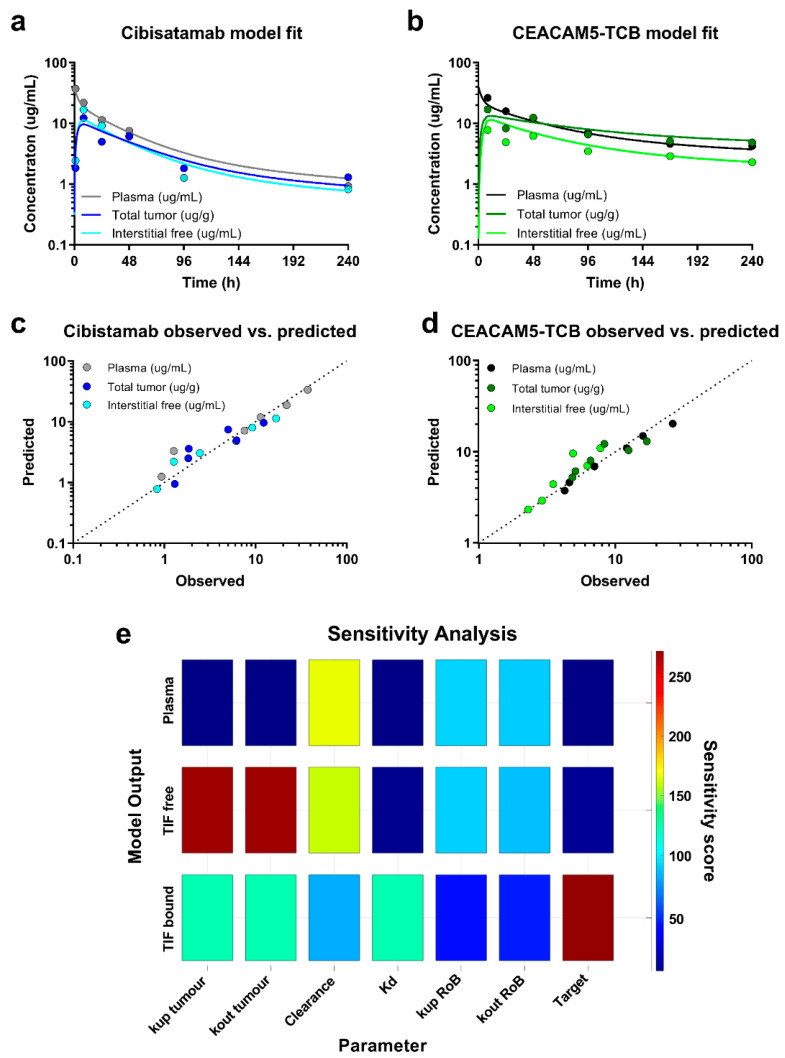
Model fitting and model performance assessment: Model fit to mean PK data measured in plasma-, total tumour tissue- and free interstitial fluid samples after a dose of 2.5 mg/kg for (**a**) cibisatamab and (**b**) CEACAM5-TCB. Further assessment of model performance based on observed vs. predicted plots for (**c**) cibisatamab and (**d**) CEACAM5-TCB and (**e**) a local sensitivity analysis for fitted PK readouts on estimated parameter (representatively shown for CEACAM5-TCB).

For both compounds, the model shows a good fit to the plasma, free interstitial tumour and total tumour PK data, which is further illustrated by observed vs. predicted plots (Figure 8a–d). All underlying estimated parameter values and corresponding estimation accuracy (%CI) can be found in Table 1. The relatively low %CI values indicate an overall good estimate of the underlying parameters. The sensitivity of the fitted model outputs on estimated parameter values is presented in Figure 8e. Our PK data allowed for a separate estimation of the tumour uptake rate (kup tumour) and the tumour outflow rate (kout tumour) for the TCBs, which were estimated to be 0.019 and 0.539 respectively. It is of note that also the Kd-values for the two TCBs were re-estimated in order to enable capturing the observed total, bound and free antibody concentration in the tumour. The newly estimated binding affinity values were ~70.5 nM and 26.4 nM for CEACAM5-TCB and cibisatamab respectively, which is markedly higher than the values reported based on SPR-measurements. This final model framework enables now a first simulation or prediction of an expected antibody PK in plasma, tumour and other tissues of interest.

## 4. Discussion

In this work, we combined a single-dose PKPD study with a tissue centrifugation methodology with the main objective to compare the distribution of two TCBs with different binding affinities to the same tumour target and evaluate how this affects their distribution to the tumour interstitial space. Based on the generated PK data, we built a mathematical model that enables the quantification of key parameters for tumour uptake and accumulation of the two TCBs and prediction of anticipated total tumour drug concentration, as well as free and bound concentration in TIF. We further compare subsequent tumour growth inhibition and local and systemic cytokine release under TCB treatment.

Our results show high TCBs uptake and accumulation in the tumour interstitial space, indicated by a total interstitial: plasma concentration ratio of 3- and 5-fold for cibisatamab and CEACAM5-TCB respectively. We estimated that approximately 65% of cibisatamab and 80% of CEACAM5-TCB reaching the tumour interstitial space is bound to the target. As such, target-binding properties play a major role in tumour accumulation, as has previously been discussed for cibisatamab [31]. Model simulations indicated that this total interstitial:plasma concentration ratio would only be ~0.5 fold if not binding to the tumour target. This is in line with prior observations in the interstitial fluid of other tissues (e.g., skin, muscle, tendon) [14]. Several aspects have to be considered in light of the high tumour uptake for the TCBs, which we report here. The tumours in this study represent well-perfused subcutaneous tumours in mice of about 250 mm^3^. Necrotic and less perfused areas, which predominantly occur at higher tumour size, will be less accessible for antibodies. In addition, the binding site barrier can limit the penetration of antibodies into deeper tissue layers. Both aspects could cause an inhomogeneous antibody distribution in the tumour [32,33,34]. Moreover, if the TCBs bind with high affinity to immune cells in the systemic circulation or in highly accessible tissues, it may limit antibody uptake to the tumour by reducing the concentration of free antibody in the circulation [34]. On the other hand, high affinity binding to tumour infiltrated immune cells could lead to additional accumulation in tumour. However, CD3-bispecifics are often designed to have high affinity to the tumour target with lower CD3 affinity in order to achieve higher tumour specificity [35,36]. In addition, in the current study, target expression was not assessed, and it was assumed that it will be constant over time. However, it is expected that in cases with target up- or down modulation, the relative unbound concentration in the interstitium may be altered accordingly.

The centrifugation method grants direct access TIF and allows for a more granular description of concentrations at the target site. It has recently been reported that published interstitial concentrations were higher based on the centrifugation method than measurements with microdialysis or back-calculated based on perfused tissue data [12]. Wiig and Swartz have previously published a thorough review around methods to isolate interstitial fluid from tissues, including microdialysis. Importantly, the centrifugation method yields comparable interstitial:plasma ratios as measurements in prenodal lymph, which is a surrogate for tissue interstitial fluid [11]. Deriving interstitial tissue concentration based on total tissue concentrations relies on the use of the interstitial fluid fraction in the respective tissue, which was measured using radiotracer studies with small molecules. This can lead to an overestimation of the apparent interstitial distribution volume for antibodies, which are excluded from a fraction of the interstitial space by steric or static hindrance by the extracellular matrix [25]. In turn, this would lead to an underestimation of the antibody interstitial concentration. If the values for the respective tissue are known, this can be corrected for by taking into account the accessible interstitial volume fraction for antibodies [14]. On the other hand, the calculation assumes no relevant intracellular uptake of the respective antibody. In cases of a high intracellular uptake of intact antibodies, this would cause an overestimation of the interstitial concentration.

Based on the biodistribution data, a tumour uptake model was constructed, which allows simulating PK readouts and quantifying underlying antibody distribution parameters. We decided to link the tumour uptake model to a PBPK model, which was previously published based on antibody PK data in the interstitial space of healthy tissues [14]. This structure provides a framework, which enables simulation of exposure in tumour and healthy tissues of interest, while considering different binding properties, expression levels or doses. This might be of interest when a target is also expressed in healthy tissues, potentially leading to undesired on-target off-tumour toxicity. Alternatively, the tumour uptake model can be linked for simplicity to a 2-compartment model, which is well calibrated to the respective plasma PK. Our model demonstrated a good fit of the PK in plasma and tumour. The estimated plasma clearance was higher for cibisatamab (0.042 mL/h) as compared to CEACAM5-TCB (0.015 mL/h). The tumour volume was fixed to 250 μL in accordance with the data, which indicate the the tumour volume remained close to this value under treatment for both TCBs (except the 10-day time point for cibisatamab). A change of tumour volume over time could theoretically lead to a dilution effect, which is also highly dependent on how the change in volume affects the tumour microenvironment and would need to be investigation over a dynamic range of tumour volume changes. For studies with longitudinal changes in tumour volume, it is suggested to reparametrize the model and account for the dynamic changes in tumour volume accordingly. Binding affinity and target expression levels were further estimated based on the data to permit a good description of bound and free antibody concentrations in the tumour. Target expression typically varies between tumour types and across patients and will need to be cautiously readjusted for other situations. The binding affinity is compound specific and usually assessed early in drug discovery. However, we showed in this case, that estimated Kd values for both TCBs were substantially higher than reported values based on SPR measurements (70.5 vs. 16 nM for cibisatamab and 26.4 vs. 0.09 nM for CEACAM5-TCB, respectively). Interestingly, recently published affinity values based on fluorescence resonance energy transfer (FRET) indicate a Kd of 48.6 nM for cibisatamab and 13.1 nM for CEACAM5-TCB [28], which are considerably closer to the model-estimated values.

CEACAM5-TCB induced higher cytokine release in TIF as compared to cibisatamab. The strongest cytokine release is anticipated to occur at the target site, upon binding to both targets in TIF. Indeed, systemic cytokine levels were much lower than corresponding local cytokine levels, indicating a low contribution of cytokines released in the tumour to elevated plasma cytokine levels. In this humanised mouse model, the target expression is restricted to the subcutaneous tumour, with no other tissues expressing CEA. Furthermore, this mouse model does not include the human innate, myeloid immune compartment, which is a contributor to the cytokine response [37,38]. Thus, using a different mouse model with respect to immune cell engraftment or target expression can have an impact on cytokine release.

We further compared the cytokine release in vitro and in humanised mice, which are two common experimental models for preclinical cytokine testing. Interestingly, for most of the cytokines the kinetics was very similar in vitro and in vivo in TIF. This indicates that immune cells present in both models similarly act as local sources and sink, leading to production and degradation of cytokines [39]. In order to compare the cytokine release in context with drug exposure, we compared TIC with the in vitro concentration, as both represent the concentration at the target site, including free and bound antibodies. In our opinion, TIC can thus be a useful readout in order to compare the two experimental systems. In terms of TIC, after a dose of 2.5 mg/kg, the observed Cmax values were 208 nM for cibisatamab and 288 nM for CEACAM5-TCB in vivo, while the highest tested in vitro concentrations were 100 nM and 20 nM for cibisatamab and CEACAM5-TCB respectively. Despite the higher in vivo Cmax, lower cytokine levels were generally observed in TIF as compared to in vitro, with the only exception of IL10 (Figure 7). Several differences between in vitro and in vivo might affect cytokine release in those systems and explain those differences. The E:T ratio (PBMC: tumour target cell number) in vitro was 10:1 while for in vivo this ratio will probably be considerably lower [40]. In both systems, this ratio depends on subsequent T-cell recruitment and proliferation upon TCB treatment. Additionally, more immuno-suppressing factors are expected in vivo, and the higher level of IL10 in vivo could be an indication of this.

In light of the data presented here, several aspects would be highly complementary in future research. Testing a dose range would allow a more detailed characterisation of the PKPD relationships and target binding saturation. The model can be further expanded to predict the impact of tumour growth on TIF or to assess any other pathophysiological changes during tumour progression (e.g., alteration of target expression) on local drug concentration with additional follow-up studies. As a next step, it is suggested to link the expanded PBPK model to the drug effect on tumour growth. With such a mechanism-based PKPD model, the drug effect can be predicted based on the local drug concentration as a result of target expression as well as binding affinity. It would be suited for compound selection as well as selection and prediction of optimal dose and dosing schedule. Furthermore, the measurement of immune cell dynamics would add mechanistic insights into their role in the cytokine response. Investigating additional compounds in a similar setting would help to elaborate on potential variability and differences in between IgG-based molecules. Together, those aspects would allow mechanistically expanding the mathematical model and moving towards more of a quantitative systems pharmacology approach. Unfortunately, those aspects were out of scope in this work due to the terminal nature of the measurements and thus very high number of humanised mice required to obtain samples at all defined time points. Therefore, we decided here to focus on the comparison of two TCBs with different binding affinities in terms of their biodistribution to the tumour interstitial space, to quantify relevant parameters driving antibody tumour distribution with a mathematical model and to characterise the elicited tumour growth inhibition and cytokine release.

## 5. Conclusions

In summary, we measured for the first time directly the TCB concentration levels at the target site in isolated interstitial fluid from tumour and show a high tumour uptake of TCBs. We provide a model, which allows simulations of interstitial TCB concentrations in tumour and other tissues considering target-binding properties and discuss total interstitial concentration (TIC) as suitable readout for comparing in vitro to in vivo PD effects. As such, the provided work improves the current understanding and model-based predictions of tumour distribution, which is an important translational aspect for tumour-targeted antibodies. The findings and discussions presented here can have important implications to support the development and preclinical testing of targeted antibody therapeutics.

## Figures and Tables

**Table 1:** Parameter estimates of the tumour uptake model.

Parameter	Estimate	%CI
Vpla (mL)	1.00	0.01
kup RoB (1/h)	0.019	0.46
kout RoB (1/h)	0.007	19
kup tumour (1/h)	0.019	3.8
kout tumour (1/h)	0.539	0.12
Target (nM)	270.1	0.12
Kd Cibisatamab (nM)	70.5	6.0
Kd CEACAM5-TCB (nM) CL cibisatamab (mL/h) CL CEACAM5-TCB (mL/h)	26.4 0.042 0.016	0.5 33.7 24.2
fVpla tumour	0.0086 ^a^	-
fVint tumour	0.30 ^a^	-

**^a^** Fixed to measured values. Vpla: plasma volume; kup tumour: uptake rate tumour, kout: outflow rate tumour, RoB: rest of body; Kd: equilibrium dissociation constant; CL: clearance; fVpla tumour: residual plasma volume fraction in harvested tumour tissue; fVint tumour: interstitial volume fraction in tumour tissue.

## Data Availability

The data that support the findings of this study are available from the corresponding author upon reasonable request. Some data may not be made available because of privacy or ethical restrictions. https://www.mdpi.com/ethics (9 November 2021). You might choose to exclude this statement if the study did not report any data.

## Supplementary Materials

The following are available online at https://www.mdpi.com/article/10.3390/pharmaceutics13122105/s1. Table S1: Overview of experimental strategy, study setup and purpose. Supplementary material S1: Indirect 125Iodine-labelling of cibisatamab and CEACAM5-TCB. Figure S1: Quality control to ensure retention of the biological activity after labelling of the TCBs with ^125^Iodine. Figure S2: PBPK model simulations before and after adaptions. Supplementary material S2: Simbiology PBPK model equations and definitions. Supplementary Table S2: Parameter definitions.

## References

[1] Yu L., Wang J.H. (2019). T cell-redirecting bispecific antibodies in cancer immunotherapy: Recent advances. J. Cancer Res. Clin..

[2] Ferl G.Z., Theil F.P., Wong H. (2016). Physiologically based pharmacokinetic models of small molecules and therapeutic antibodies: A mini-review on fundamental concepts and applications. Biopharm. Drug Dispos..

[3] Wang W., Wang E.Q., Balthasar J.P. (2008). Monoclonal antibody pharmacokinetics and pharmacodynamics. Clin. Pharmacol. Ther..

[4] Ovacik M., Lin K.D. (2018). Tutorial on monoclonal antibody pharmacokinetics and its considerations in early development. CTS-Clin. Transl. Sci..

[5] Ryman J.T., Meibohm B. (2017). Pharmacokinetics of monoclonal antibodies. CPT Pharmacomet. Syst. Pharmacol..

[6] Xenaki K.T., Oliveira S., van Bergen En Henegouwen P.M.P. (2017). Antibody or antibody fragments: Implications for molecular imaging and targeted therapy of solid tumors. Front. Immunol..

[7] Boswell C.A., Bumbaca D., Fielder P.J., Khawli L.A. (2012). Compartmental tissue distribution of antibody therapeutics: Experimental approaches and interpretations. Aaps J..

[8] Dudal S., Hinton H., Giusti A.M., Bacac M., Muller M., Fauti T., Colombetti S., Heckel T., Giroud N., Klein C. (2016). Application of a mabel approach for a t-cell-bispecific monoclonal antibody: Cea tcb. J. Immunother..

[9] Harper J., Adams K.J., Bossi G., Wright D.E., Stacey A.R., Bedke N., Martinez-Hague R., Blat D., Humbert L., Buchanan H. (2018). An approved in vitro approach to preclinical safety and efficacy evaluation of engineered t cell receptor anti-cd3 bispecific (immtac) molecules. PLoS ONE.

[10] Wiig H., Tenstad O., Iversen P.O., Kalluri R., Bjerkvig R. (2010). Interstitial fluid: The overlooked component of the tumor microenvironment?. Fibrogenesis Tissue Repair..

[11] Wiig H., Swartz M.A. (2012). Interstitial fluid and lymph formation and transport: Physiological regulation and roles in inflammation and cancer. Physiol. Rev..

[12] Chang H.P., Kim S.J., Shah D.K. (2020). Whole-body pharmacokinetics of antibody in mice determined using enzyme-linked immunosorbent assay and derivation of tissue interstitial concentrations. J. Pharm. Sci..

[13] Wiig H., Aukland K., Tenstad O. (2003). Isolation of interstitial fluid from rat mammary tumors by a centrifugation method. Am. J. Physiology. Heart Circ. Physiol..

[14] Eigenmann M.J., Karlsen T.V., Krippendorff B.F., Tenstad O., Fronton L., Otteneder M.B., Wiig H. (2017). Interstitial igg antibody pharmacokinetics assessed by combined in vivo- and physiologically-based pharmacokinetic modelling approaches. J. Physiol..

[15] Haslene-Hox H., Oveland E., Berg K.C., Kolmannskog O., Woie K., Salvesen H.B., Tenstad O., Wiig H. (2011). A new method for isolation of interstitial fluid from human solid tumors applied to proteomic analysis of ovarian carcinoma tissue. PLoS ONE.

[16] Bacac M., Klein C., Umana P. (2016). Cea tcb: A novel head-to-tail 2:1 t cell bispecific antibody for treatment of cea-positive solid tumors. Oncoimmunology.

[17] Bacac M., Fauti T., Sam J., Colombetti S., Weinzierl T., Ouaret D., Bodmer W., Lehmann S., Hofer T., Hosse R.J. (2016). A novel carcinoembryonic antigen t-cell bispecific antibody (cea tcb) for the treatment of solid tumors. Clin. Cancer Res..

[18] Hammarstrom S. (1999). The carcinoembryonic antigen (cea) family: Structures, suggested functions and expression in normal and malignant tissues. Semin. Cancer Biol..

[19] Shultz L.D., Lyons B.L., Burzenski L.M., Gott B., Chen X., Chaleff S., Kotb M., Gillies S.D., King M., Mangada J. (2005). Human lymphoid and myeloid cell development in nod/ltsz-scid il2r gamma null mice engrafted with mobilized human hemopoietic stem cells. J. Immunol..

[20] Rongvaux A., Takizawa H., Strowig T., Willinger T., Eynon E.E., Flavell R.A., Manz M.G. (2013). Human hemato-lymphoid system mice: Current use and future potential for medicine. Annu. Rev. Immunol..

[21] Stripecke R., Munz C., Schuringa J.J., Bissig K.D., Soper B., Meeham T., Yao L.C., Di Santo J.P., Brehm M., Rodriguez E. (2020). Innovations, challenges, and minimal information for standardization of humanized mice. EMBO Mol. Med..

[22] Wiig H., Tenstad O., Bert J.L. (2005). Effect of hydration on interstitial distribution of charged albumin in rat dermis in vitro. J. Physiol..

[23] Fraker P.J., Speck J.C. (1978). Protein and cell membrane iodinations with a sparingly soluble chloroamide, 1,3,4,6-tetrachloro-3a,6a-diphrenylglycoluril. Biochem. Biophys. Res. Commun..

[24] Parving H.H., Gyntelberg F. (1973). Albumin transcapillary escape rate and plasma volume during long-term beta-adrenergic blockade in essential hypertension. Scand. J. Clin. Lab. Investig..

[25] Wiig H., Gyenge C.C., Tenstad O. (2005). The interstitial distribution of macromolecules in rat tumours is influenced by the negatively charged matrix components. J. Physiol..

[26] Fronton L., Pilari S., Huisinga W. (2014). Monoclonal antibody disposition: A simplified pbpk model and its implications for the derivation and interpretation of classical compartment models. J. Pharmacokinet Pharmacodyn..

[27] Garg A. (2007). Investigation of the Role of Fcrn in the Absorption, Distribution and Elimination of Monoclonal Antibodies.

[28] Van De Vyver A.J., Weinzierl T., Eigenmann M.J., Frances N., Herter S., Buser R.B., Somandin J., Diggelmann S., Limani F., Lehr T. (2021). Predicting tumor killing and t-cell activation by t-cell bispecific antibodies as a function of target expression: Combining in vitro experiments with systems modeling. Mol. Cancer Ther..

[29] Boswell C.A., Mundo E.E., Ulufatu S., Bumbaca D., Cahaya H.S., Majidy N., Van Hoy M., Schweiger M.G., Fielder P.J., Prabhu S. (2014). Comparative physiology of mice and rats: Radiometric measurement of vascular parameters in rodent tissues. Mol. Pharm..

[30] Schmidt M.M., Wittrup K.D. (2009). A modeling analysis of the effects of molecular size and binding affinity on tumor targeting. Mol. Cancer Ther..

[31] Lehmann S., Perera R., Grimm H.P., Sam J., Colombetti S., Fauti T., Fahrni L., Schaller T., Freimoser-Grundschober A., Zielonka J. (2016). In vivo fluorescence imaging of the activity of cea tcb, a novel t-cell bispecific antibody, reveals highly specific tumor targeting and fast induction of t-cell-mediated tumor killing. Clin. Cancer Res..

[32] Fujimori K., Covell D.G., Fletcher J.E., Weinstein J.N. (1990). A modeling analysis of monoclonal antibody percolation through tumors: A binding-site barrier. J. Nucl. Med..

[33] Singh A.P., Guo L., Verma A., Wong G.G., Thurber G.M., Shah D.K. (2020). Antibody coadministration as a strategy to overcome binding-site barrier for adcs: A quantitative investigation. AAPS J..

[34] Saga T., Neumann R.D., Heya T., Sato J., Kinuya S., Le N., Paik C.H., Weinstein J.N. (1995). Targeting cancer micrometastases with monoclonal antibodies: A binding-site barrier. Proc. Natl. Acad. Sci. USA.

[35] Vafa O., Trinklein N.D. (2020). Perspective: Designing t-cell engagers with better therapeutic windows. Front. Oncol.

[36] Bortoletto N., Scotet E., Myamoto Y., D’Oro U., Lanzavecchia A. (2002). Optimizing anti-cd3 affinity for effective t cell targeting against tumor cells. Eur J. Immunol..

[37] Claus C., Ferrara C., Xu W., Sam J., Lang S., Uhlenbrock F., Albrecht R., Herter S., Schlenker R., Husser T. (2019). Tumor-targeted 4-1bb agonists for combination with t cell bispecific antibodies as off-the-shelf therapy. Sci. Transl. Med..

[38] Godbersen-Palmer C., Coupet T.A., Grada Z., Zhang S.C., Sentman C.L. (2020). Toxicity induced by a bispecific t cell-redirecting protein is mediated by both t cells and myeloid cells in immunocompetent mice. J. Immunol..

[39] Hart Y., Reich-Zeliger S., Antebi Y.E., Zaretsky I., Mayo A.E., Alon U., Friedman N. (2014). Paradoxical signaling by a secreted molecule leads to homeostasis of cell levels. Cell.

[40] Betts A., Haddish-Berhane N., Shah D.K., van der Graaf P.H., Barletta F., King L., Clark T., Kamperschroer C., Root A., Hooper A. (2019). Correction to: A translational quantitative systems pharmacology model for cd3 bispecific molecules: Application to quantify t cell-mediated tumor cell killing by p-cadherin lp dart(r). AAPS J..

