# Supplementary material for: Pharmacokinetics and Pharmacodynamics of T-Cell Bispecifics in the Tumour Interstitial Fluid
Source: Pharmaceutics. 2021 Dec 7;13(12):2105. doi: 10.3390/pharmaceutics13122105 (PMC8705663; doi:10.3390/pharmaceutics13122105)
Supplement: Supplementary file 1 [file pharmaceutics-13-02105-s001.zip › pharmaceutics-1479569-supplementary.pdf]

Supplementary Materials: Pharmacokinetics and Pharmacodynamics of T-Cell Bispecifics in the Tumour Interstitial Fluid

Miro Julian Eigenmann, Tine Veronica Karlsen, Marek Wagner, Olav Tenstad, Tina Weinzierl, Tanja Fauti, Hans Peter Grimm, Trude Skogstrand, Christian Klein, Johannes Sam, Pablo Umana, Marina Bacac, Helge Wiig and Antje-Christine Walz

Table S1. Overview of experimental strategy, study setup and purpose

Study	Study Setup	Purpose & Notes
Tumour engraftment[a]	• Animals: huNSG CD34+ mice (n = 45)<br>• Injected cells: MKN45 tumour cells<br>• Location: subcutaneous back engraftment	• Tumour bearing animals required for biodistribution study
**Radiolabelling[a]**	• **Human serum albumin:** labelled with ^{125}I using iodogen method<br>• **T cell bispecifics:** labelled with ^{125}I using indirect labelling approach	• ^{125}I-HSA and ^{125}I-TCBs used in radiotracer- and biodistribution study respectively<br>• Note: Indirect labelling of TCBs used to avoid functional impairment due to labelling
**Radiotracer study[a]**	• **Animals:** NSG mice (n = 13)<br>• **Samples:** skin and tumour (total tissue and centrifuged)<br>• **Sampling time:** 14, 17 and 20 days post-engraftment<br>• **Tracers:** ^{51}Cr-EDTA as extracellular- and ^{125}I-HSA as intravascular tracer<br>• **Tracer infusion time:** 1 hour (^{51}Cr-EDTA) and 5 min (^{125}I-HSA) before tissue sampling	• Evaluate centrifugation method to derive interstitial fluid sample from tumour (skin as positive control)<br>• Assess impact of tumour size on TIF sample<br>• Investigate composition of total tumour sample (e.g., interstitial fluid-, residual plasma fraction)<br>• Note: Confirmation of deriving a relevant TIF sample by centrifugation required
**Biodistribution study[b]**	• **Animals:** huNSG CD34+ mice (n = 45)<br>• **Samples:** plasma and tumour tissue and TIF<br>• **Time points:** 1, 8, 24, 48, 96 & 240 h<br>• **Treatment groups:** control, 2.5 mg*kg^{-1} cibisatamab or CEACAM5-TCB<br>• **PK measurement:** plasma, total tumour tissue and TIF by γ-counting<br>• **PD assessment:** (1) tumour volume by caliper measurement, (2) cytokine levels in plasma and TIF by Magnetic Luminex Performance Assay	• Measure PK in plasma, total tumour tissue and TIF for two TCBs with different target binding affinity<br>• Compare systemic plasma concentration levels to local drug concentration levels in TIF (free, bound and total)<br>• Monitor and compare PD effects of both TCBs under treatment (i.e., tumour growth inhibition and local versus systemic cytokine levels)<br>• Compare cytokine release in TIF to cytokine release in a corresponding in vitro setting

**Modelling**[b]	• **Software:** Matlab and Simbiology<br>• **Fitted data:** plasma, total tumour and free interstitial PK for both TCBs<br>• **Model structure:** simple tumour uptake model linked to a reduced PBPK model<br>• **Estimated parameters:** plasma volume, plasma clearance, uptake and removal rate tumour, uptake and removal rate rest of body, target expression in tumour, target binding affinity<br>• **Model assessment:** parameter accuracy (as %CI), observed vs. predicted plots and sensitivity analysis	• Quantify key parameters for tumour distribution and accumulation<br>• Allow simulation of tumour PK readouts (total tumour and free, bound and total interstitial concentrations) and quantify the impact of target binding on tumour accumulation<br>• The tumour uptake model to a reduced PBPK model and provides a framework to simultaneously simulate TCB uptake in tumour and other tissues of interest

[a]Studies, which were performed in preparation and prerequisite of the main work (grey background)
[b]All work packages conducted during the main experiments (white background)

Supplementary material S1: Indirect ^{125}I-labelling of cibisatamab and CEACAM5-TCB.

In order to avoid impact of the labelling procedure on the pharmacological activity, the two T-cell bispecific antibodies were labelled by an indirect labelling approach. During this indirect labelling approach, $^{125}I^-$ ions were first activated to $^{125}I^+$ ions in a Pierce™ Pre-Coated Iodination tube (Thermo Scientific™ Catalogue number 28601) according to the manufacturer's recommendations. Activated ^{125}I was then immediately transferred into another tube containing the protein for electrophilic attack of the ortho ring position of tyrosine. Cibisatamab (9 µL) and CEACAM5-TCB (93 µL) were added to the bottom of separate 1.5 mL screw cap tubes and diluted to a total volume of 100 µL by addition of Tris iodination buffer (25 mM Tris-HCl, pH 7.5, 0.4 M NaCl) at room temperature, i.e., about 1 nmole of each protein to be iodinated. One iodination tube was washed with 1 ml Tris iodination buffer, decanted and 100 µL buffer plus 10 µL (37 MBq) carrier free Na ^{125}I (PerkinElmer NEZ033A, specific activity of 643 GBq/mg I) added to the bottom of the tube. Activation of ^{125}I (oxidation to $^{125}I^+$) was allowed for 6 minutes during gentle agitation before transferring to the 1.5 mL tube containing the respective antibody. Iodination of the tyrosine moieties of the antibody was allowed for 8 min during gentle agitation. The reaction was then stopped by addition of 50 µL scavenger buffer (10 mg tyrosine/mL in Tris iodination buffer, pH 7.4) for 5 minutes. Finally, 1 ml of storage buffer (1.0 mL 25 mM Tris-HCl, pH 7.5, 0.4 M NaCl, 5 mM EDTA, 0.05% sodium azide) was added and the antibody stock solution was stored in the refrigerator at 4 °C until use.

Figure S1: Quality control to ensure retention of the biological activity after labelling of the TCBs with ^{125}Iodine.

For both labelling methods, the retention of the biological activity of the two antibodies after ^{125}I-labelling was tested. The T-cell activation capability was compared for the respective labelled and unlabelled antibodies using a T-cell activation bioassay kit (JURKAT NFAT from Promega). The assay was performed according to the provider's instruction manual. In brief, 40,000 MKN45 tumour cells (in 25 µL) and 100,000 JURKAT NFAT cells (in 25 µL) were added to each well in a white-walled 96-well plate. For cibisatamab seven different concentrations ranging from 13 to 200,000 pM were added in triplicates and for CEACAM5-TCB seven different concentrations ranging from 6 to 100,000 pM were used. A triplicate of wells containing untreated cells was used for background correction. The same concentrations were tested for the labelled and unlabelled version of the T-cell bispecific antibodies. After adding the antibody dilutions to the corresponding wells, the plates were incubated for 6 hours at 37 °C in 5% CO_2. Afterwards, 100 µL of a

1:1 mixture of Bio-Glo and assay mix was added to each well and the plates were incubated for another 10 min in the dark at room temperature. Eventually, the luminescence in the wells were measured using a Perkin Elmer Multilabel counter. After labelling with an indirect labelling approach, no impact on activity in the JURKAT NFAT assay was observed when compared with the unlabelled antibodies, whereas, labelling by the iodogen method led to a significant decrease in functional activity for both TCBs (Figure S1). Based on these results, the TCBs were labelled by the indirect approach for all further studies.

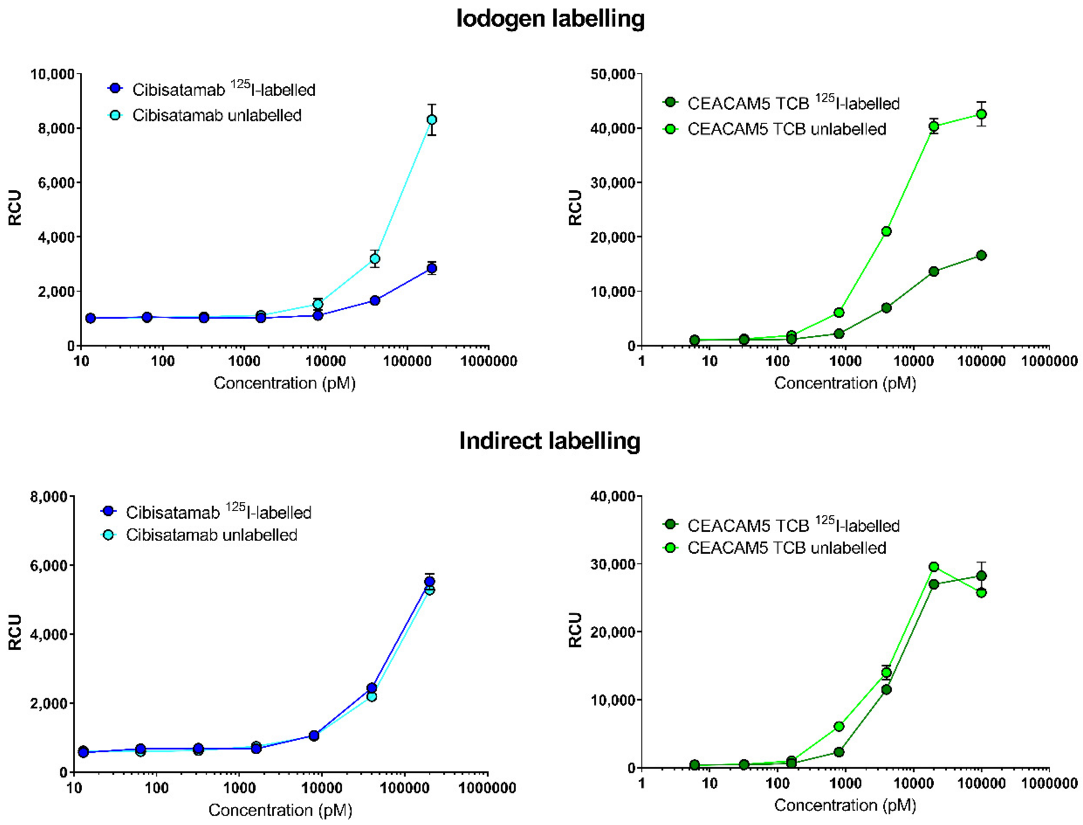

Figure S1. Activity of labelled versus unlabelled cibistamab (blue) and CEACAM5-TCB (green) in the NFAT Jurkat assay when ^{125}I-labelling was performed by the iodogen approach (upper panel) or by the indirect labelling approach (lower panel). Data are presented as mean ± SD (n = 3).

Before the start of each experiment, any potentially unbound ^{125}I was removed from solution using Amicon® Ultra-4 Centrifugal Filter Units (50 kDa cut-off) and buffer exchange. Furthermore, size exclusion chromatography was used for further testing of the purity of the injection solution containing the labelled compounds.

Figure S2. PBPK model simulations before and after adaptions.

The original PBPK model published by Eigenmann et al. [1], which was used as bases of the PBPK approach in this work, has been simplified in two aspects. (1) all tissue plasma spaces were lumped together with the systemic plasma space, (2) blood flows were removed from the PBPK model as they have previously been shown, by a sensitivity analysis, to have no relevant impact on the antibody biodistribution and consequently the concentration outputs of the PBPK model (1). In order to confirm that there was no impact on simulated concentrations with the PBPK model, model simulations for plasma and skin (as example tissue) after a dose of 10 mg/kg were performed, overlaid before

and after the mentioned changes (Figure S2).

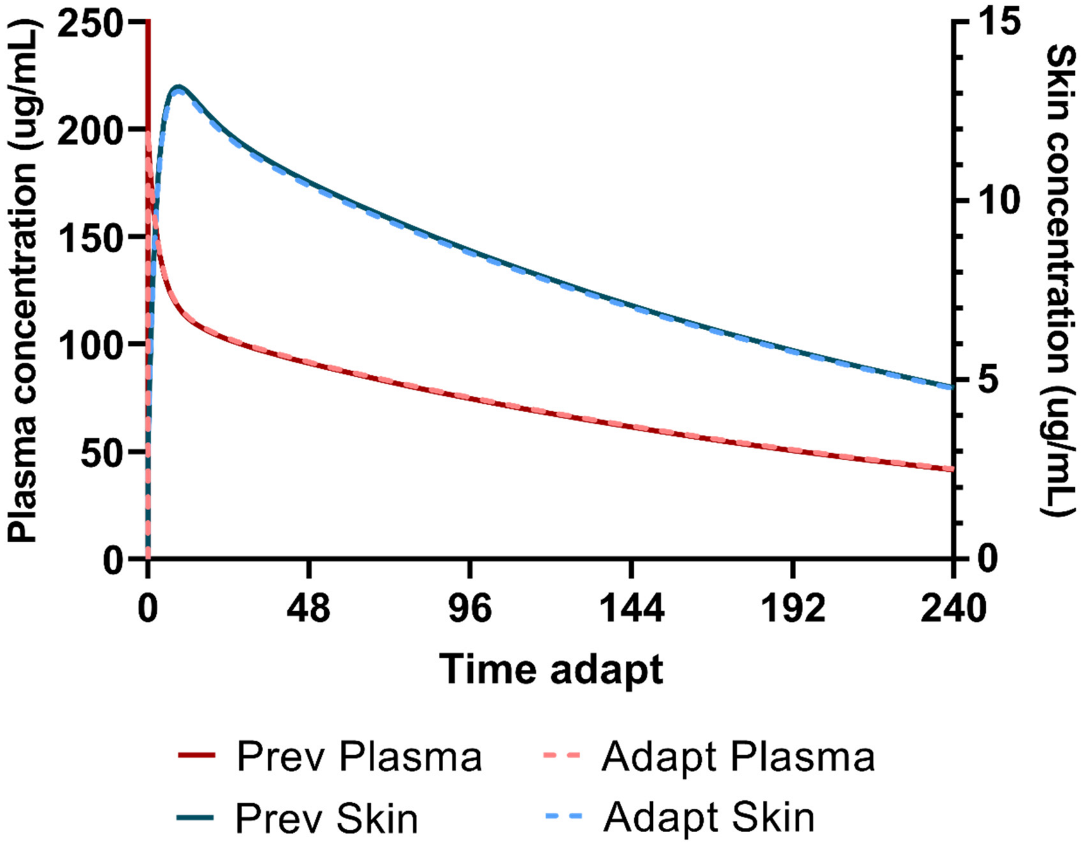

Figure S2. Overlay of model simulations for plasma and skin concentrations before (solid lines) and after (dashed lines) lumping the plasma spaces of the model and removing the blood flows from the model. Skin was simulated as example for total tissue concentrations. The resulting simulations confirm that there was no difference in the simulated model outputs. Simulations were performed for a dose of 10 mg/kg.

Supplementary material S2: Simbiology PBPK model equations and definitions.

Equations and definitions of the tumour uptake and PBPK model, which were used in the Simbiology model are reported below. For more background information and details around parameter values of the linked PBPK model, please refer to the original publication (1). Estimated and measured parameter values for plasma, tumour and rest of body are reported in the main body of this work.

Table S2. Parameter definitions.

Apla = antibody amount in plasma	Cpla = antibody concentration in plasma
Ai = antibody amount in interstitium	Aif = antibody amount free in interstitium
Aib = antibody amount bound in interstitium	V = total tissue volume
Vi = interstitial tissue volume	kup = antibody uptake rate
kout = antibody outflow rate	CLint = tissue intrinsic clearance
targ = tot. target amount	fCL = fractional contribution to clearance
$targ_f$ = free target amount	fVv = residual plasma fraction
fVi = interstitial volume fraction	fexcl = excluded volume fraction
koff = antibody binding off-rate	kon = antibody binding on-rate

Plasma

$$\frac{d(Apla)}{dt} = -(Kup_{bon} * \frac{Apla}{Vpla}) + (kout_{bon} * Ai_{bone}/Vibon) - (Kup_{lun} * \frac{Apla}{Vpla}) + (kout_{lun} * \frac{Ai_{lung}}{Vilun}) - (Kup_{ski} * \frac{Apla}{Vpla})$$
$$+ (kout_{ski} * \frac{Ai_{skin}}{Viski}) - (Kup_{mus} * \frac{Apla}{Vpla}) + (kout_{mus} * \frac{Ai_{muscle}}{Vimus}) - (Kup_{gut} * \frac{Apla}{Vpla}) + (kout_{gut}$$
$$* Ai_{gitract}/Vigut) + (kout_{kid} * \frac{Ai_{kidney}}{Vikid}) - (Kup_{hea} * \frac{Apla}{Vpla}) + (kout_{hea} * \frac{Ai_{heart}}{Vihea}) - (Kup_{adi} * \frac{Apla}{Vpla})$$
$$+ (kout_{bra} * \frac{Ai_{brain}}{Vibra}) + (kout_{adi} * \frac{Ai_{adipose}}{Viadi}) - (\frac{Apla}{Vpla} * CLint_{pla} * fCL_{lun}) - (\frac{Apla}{Vpla} * CLint_{pla}$$
$$* fCL_{hea}) - (\frac{Apla}{Vpla} * CLint_{pla} * fCL_{mus}) - (\frac{Apla}{Vpla} * CLint_{pla} * fCL_{bon}) - (\frac{Apla}{Vpla} * CLint_{pla} * fCL_{ski})$$
$$- (\frac{Apla}{Vpla} * CLint_{pla} * fCL_{spl}) - (\frac{Apla}{Vpla} * CLint_{pla} * fCL_{liv}) - (\frac{Apla}{Vpla} * CLint_{pla} * fCL_{gut}) - (\frac{Apla}{Vpla}$$
$$* CLint_{pla} * fCL_{kid}) - (\frac{Apla}{Vpla} * CLint_{pla} * fCL_{adi}) - (\frac{Apla}{Vpla} * CLint_{pla} * fCL_{bra}) - (Kup_{tum} * Apla)$$
$$+ (Kout_{tum} * Aif_{tumor}) - (Kup_{RoB} * Apla) + (kout_{RoB} * A_{RoB})$$

$$Ctis_{plasma} = \frac{Apla}{Vpla}$$

<u>Rest of Body</u>

$$\frac{d(A_{RoB})}{dt} = (Kup_{RoB} * Apla) - (kout_{RoB} * A_{RoB})$$

Tumour

$$\frac{d(Aif_{tumour})}{dt} = (Kup_{tum} * Apla) - (Kout_{tum} * Aif_{tumour}) - ((Aif_{tumor}/Vitum) * kon * targ_f * Vitum) + (koff * Aib_{tumour})$$

$$\frac{d(Aib_{tumour})}{dt} = ((Aif_{tumor}/Vitum) * kon * targ_f * Vitum) - (koff * Aib_{tumour})$$

$$Cint_{tumor_{bound}} = \frac{Aimb_{tumour}}{Vitum}$$

$$Cint_{tumor_{free}} = \frac{Aif_{tumour}}{Vitum}$$

$$Cint_{tumor} = \frac{Aif_{tumor} + Aimb_{tumour}}{Vitum}$$

$$targ_f = targ - (\frac{Aimb_{tumour}}{Vitum})$$

$$Ctot_{tumour} = fVv_{tum} * Ctis_{plasma} + fVint_{tum} * Cint_{tumour}$$

Other tissues

- Bone

$$\frac{d(Ai_{bone})}{dt} = (Kup_{bon} * \frac{Apla}{Vpla}) - (kout_{bon} * Ai_{bone}/Vibon)$$

$$Ctot_{bone} = \frac{Ai_{bone} + \frac{Apla}{Vpla} * fVvbon}{Vbon}$$

$$Cint_{bone} = \frac{Ai_{bone}}{Vbon}$$

- Lung

$$\frac{d(Ai_{lung})}{dt} = (Kup_{lun} * \frac{Apla}{Vpla}) - (kout_{lun} * \frac{Ai_{lung}}{Vilun})$$

$$Ctot_{lung} = \frac{Ai_{lung} + \frac{Apla}{Vpla} * fVvlun}{Vlun}$$

$$Cint_{lung} = \frac{Ai_{lung}}{Vlun}$$

- Skin

$$\frac{d(Ai_{skin})}{dt} = (Kup_{ski} * \frac{Apla}{Vpla}) - (kout_{ski} * \frac{Ai_{skin}}{Viski})$$

$$Cint_{skin} = \frac{Ai_{skin}}{Vski}$$

$$Cint_{skin_{corr}} = (Ai_{skin} + \frac{Apla}{Vpla} * fVpla_{cent_{ski}} * Viski)/(Viski * fexcl_{ski})$$

$$Ctot_{skin} = \frac{Ai_{skin} + \frac{Apla}{Vpla} * fVvski}{Vski}$$

- Muscle

$$\frac{d(Ai_{muscle})}{dt} = (Kup_{mus} * \frac{Apla}{Vpla}) - (kout_{mus} * \frac{Ai_{muscle}}{Vimus})$$

$$Cint_{muscle} = (Ai_{muscle})/Vmus$$

$$Cint_{muscle_{corr}} = (\frac{(Ai_{muscle} + Apla)}{Vpla} * fVpla_{cent_{mus}} * Vimus)/(Vimus * fexcl_{mus})$$

$$Ctot_{muscle} = \frac{Ai_{muscle} + \frac{Apla}{Vpla} * fVvmus}{Vmus}$$

- GI tract

$$\frac{d(Ai_{gitract})}{dt} = (Kup_{gut} * \frac{Apla}{Vpla}) - (kout_{gut} * \frac{Ai_{gitract}}{Vigut})$$

$$Cint_{gut} = \frac{Ai_{gitract}}{Vgut}$$

$$Ctot_{gut} = \frac{Ai_{gitract} + \frac{Apla}{Vpla} * fVvgut}{Vgut}$$

- Heart

$$\frac{d(Ai_{heart})}{dt} = (Kup_{hea} * \frac{Apla}{Vpla}) - (kout_{hea} * \frac{Ai_{heart}}{Vihea})$$

$$Cint_{heart} = \frac{Ai_{heart}}{Vhea}$$

$$Ctot_{heart} = \frac{Ai_{heart} + \frac{Apla}{Vpla} * fVvhea}{Vhea}$$

- Adipose

$$\frac{d(Ai_{adipose})}{dt} = (Kup_{adi} * \frac{Apla}{Vpla}) - (kout_{adi} * \frac{Ai_{adipose}}{Viadi})$$

$$Cint_{adipose} = (Ai\_adipose)/Vadi$$

$$Ctot_{adipose} = (Ai\_adipose + Apla/Vpla * fVvadi)/Vadi$$

- Kidney

$$Ctot_{kidney} = \frac{Ai_{kidney} + \frac{Apla}{Vpla} * fVvkid}{Vkid}$$

- Liver

$$Ctot_{liver} = \frac{\frac{Apla}{Vpla} * fVvliv}{Vliv}$$

- Spleen

$$Ctot_{spleen} = \frac{\frac{Apla}{Vpla} * fVvspl}{Vspl}$$

- Brain

$$Ctot_{brain} = \frac{Ai_{brain} + \frac{Apla}{Vpla} * fVvbra}{Vbra}$$

References

1. Eigenmann, M.J.; Karlsen, T.V.; Krippendorff, B.F.; Tenstad, O.; Fronton, L.; Otteneder, M.B.; Wiig, H. Interstitial igg antibody pharmacokinetics assessed by combined in vivo- and physiologically-based pharmacokinetic modelling approaches. *J Physiol* **2017**, *595*, 7311-7330.
